# Supplementary material for: Current knowledge of physicians’ dual practice in Iran: A scoping review and defining the research agenda for achieving universal health coverage
Source: PLoS One. 2022 Nov 18;17(11):e0277896. doi: 10.1371/journal.pone.0277896 (PMC9674143; doi:10.1371/journal.pone.0277896)
Supplement: S1 Table — (DOCX) [file pone.0277896.s001.docx]

S1 Table. Search strategies and results for selected databases

|  | database | Date conducted | Search strategy | # results |
| --- | --- | --- | --- | --- |
| Databases Searches | PubMed | August 3, 2020 | ("dual practice*"[tiab] OR "multiple practice*"[tiab] OR "Dual occupation*"[tiab] OR "Dual career*"[ tiab] OR "Dual employment*"[ tiab] OR "Dual working"[tiab] OR "Dual job*"[tiab] OR "moonlight*"[tiab] OR “Private practice"[tiab] OR "multiple job holding”[tiab] OR "multiple job-holding”[tiab] OR "second job*”[tiab] OR "Employment"[Mesh] OR "Practice Patterns, Nurses'"[Mesh] OR "Practice Patterns, Dentists'"[Mesh] OR "Practice Patterns, Physicians'"[Mesh] OR "Personnel Staffing and Scheduling"[Mesh] OR "Physicians/economics"[Mesh] OR "Physicians/ethics"[Mesh] OR "Physicians/legislation and jurisprudence"[Mesh] OR "Physicians/organization and administration"[Mesh] OR "Physicians/supply and distribution"[Mesh] OR "Private Practice"[Mesh]) AND (Iran[Title/Abstract]) | 359 |
|  | Embase | August 3, 2020 | ('dual practice*':ti,ab,kw OR 'multiple practice*':ti,ab,kw OR 'dual job*':ti,ab,kw OR 'second job*':ti,ab,kw OR 'dual occupation*':ti,ab,kw OR 'dual career*':ti,ab,kw OR 'dual employment':ti,ab,kw OR 'dual working':ti,ab,kw OR moonlight*:ti,ab,kw OR 'multiple job holding':ti,ab,kw OR 'multiple job-holding':ti,ab,kw) AND Iran:ti,ab,kw | 14 |
|  | Web of Science core collection | August 3, 2020 | TS=(("dual practice*" OR "multiple practice*" OR "dual job*" OR "second job*” OR "dual occupation*" OR "dual career*" OR "dual employment" OR "dual working" OR moonlight* OR "multiple job holding" OR “multiple job-holding” ) AND Iran)  Indexes=SCI-EXPANDED, SSCI, A&HCI, CPCI-S, CPCI-SSH, BKCI-S, BKCI-SSH, ESCI, CCR-EXPANDED, IC Timespan=All years | 15 |
|  | Scopus | August 3, 2020 | TITLE-ABS-KEY ("dual practice*" OR "multiple practice*" OR "dual job*" OR "second job*” OR "dual occupation*" OR "dual career*" OR "dual employment" OR "dual working" OR moonlight* OR "multiple job holding" OR “multiple job-holding”) AND TITLE-ABS-KEY (Iran) | 24 |
|  | Magiran, Iranian magazines reference | August 3, 2020 | “dual practice" OR "multiple practice" OR "dual job" OR "second job” OR OR "multiple job holding" OR “multiple job-holding” | 17 |
|  | SID, Scientific Information Database | August 3, 2020 | “dual practice" OR "multiple practice" OR "dual job" OR "second job” OR OR "multiple job holding" OR “multiple job-holding” | 5 |
|  | Google scholar  https://scholar.google.com/ |  | (“dual practice" OR "multiple practice" OR "dual job" OR "second job” OR "dual occupation" OR "dual career" OR "dual employment" OR "dual working" OR moonlighting OR "multiple job holding" OR “multiple job-holding”)+(Iran) | 15 |
|  | Total |  |  | 449 |
|  | Total with duplicates removed |  |  | 403 |
